# Comparative Analysis of Virtual Screening Approaches in the Search for Novel EphA2 Receptor Antagonists

**DOI:** 10.3390/molecules200917132

**Published:** 2015-09-17

**Authors:** Donatella Callegari, Daniele Pala, Laura Scalvini, Massimiliano Tognolini, Matteo Incerti, Silvia Rivara, Marco Mor, Alessio Lodola

**Affiliations:** 1 Dipartimento di Farmacia, Università degli Studi di Parma, Parma 43124, Italy; E-Mails: donatella.callegari@studenti.unipr.it (D.C.); daniele.pala@nemo.unipr.it (D.P.); laura.scalvini@studenti.unipr.it (L.S.); massimiliano.tognolini@unipr.it (M.T.); matteo.incerti@unipr.it (M.I.); silvia.rivara@unipr.it (S.R.); marco.mor@unipr.it (M.M.); 2 Department of Applied Sciences, Northumbria University at Newcastle, Newcastle-Upon-Tyne, NE1 8ST, UK

**Keywords:** drug design, PPI inhibitors, EphA2 antagonist, UniPR129, virtual screening, shape screening, pharmacophore search, docking

## Abstract

The EphA2 receptor and its ephrin-A1 ligand form a key cell communication system, which has been found overexpressed in many cancer types and involved in tumor growth. Recent medicinal chemistry efforts have identified bile acid derivatives as low micromolar binders of the EphA2 receptor. However, these compounds suffer from poor physicochemical properties, hampering their use *in vivo*. The identification of compounds able to disrupt the EphA2-ephrin-A1 complex lacking the bile acid scaffold may lead to new pharmacological tools suitable for *in vivo* studies. To identify the most promising virtual screening (VS) protocol aimed at finding novel EphA2 antagonists, we investigated the ability of both ligand-based and structure-based approaches to retrieve known EphA2 antagonists from libraries of decoys with similar molecular properties. While ligand-based VSs were conducted using UniPR129 and ephrin-A1 ligand as reference structures, structure-based VSs were performed with Glide, using the X-ray structure of the EphA2 receptor/ephrin-A1 complex. A comparison of enrichment factors showed that ligand-based approaches outperformed the structure-based ones, suggesting ligand-based methods using the G-H loop of ephrin-A1 ligand as template as the most promising protocols to search for novel EphA2 antagonists.

## 1. Introduction

The erythropoietin-producing hepatocellular carcinoma (Eph) receptors constitute the largest family of tyrosine kinase receptors in mammals and it includes at least fourteen members, such as the EphA1–EphA8, EphA10, EphB1–B4 and EphB6 receptor subtypes [1]. The Eph receptors are activated by membrane-anchored proteins, called ephrins, which are divided in ephrinA1–A5 and ephrinB1–B3 subclasses, respectively [2]. The Eph receptors and their ephrin ligands constitute a cell-cell communication system, which is essential for the regulation of several processes during the embryonic morphogenesis [3]. Eph-ephrin system preserves the cellular architecture in various epithelia and modulates tissue renewal in the adult. A deregulation of this system, and in particular of the activity of EphA2/ephrin-A1 signaling complex, has been related to cancer insurgence and progression [4]. Indeed, the EphA2 receptor has been found overexpressed in several cancer types [5], while the inhibition of EphA2 receptor with monoclonal antibodies [6] or soluble receptors [7] has been shown to effectively suppress cancer progression and angiogenesis in animal models [8]. For these reasons [9], the EphA2/ephrin-A1 interface is currently explored as a target for the development of new antitumorigenic and antiangiogenic treatments [10,11].

**Figure 1:**
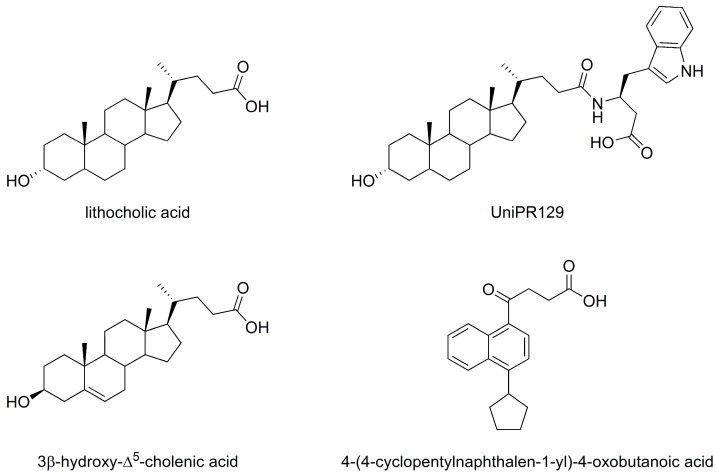
Chemical structures of selected EphA2 receptor antagonists.

A screening campaign recently conducted in our laboratories allowed the identification of small molecules able to inhibit the EphA2/ephrin-A1 interaction, including the (3α,5β)-3-hydroxycholan-24-oic acid (lithocholic acid, LCA, Figure 1, [12]) which turned out to be a competitive antagonist of the EphA2 receptor [13]. Further medicinal chemistry efforts [14,15] led to the identification of the l-β-homo-tryptophan derivative of LCA (UniPR129, Figure 1) as a potent antagonist of the EphA2 receptor, having an inhibitory constant (K_i_) of 370 nM [16]. However, this compound had modest solubility, which hampered its use *in vivo* by the oral route [17]. The identification of new compounds able to disrupt the EphA2/ephrin-A1 complex may lead to pharmacological tools featured by better physicochemical properties and thus suitable for *in vivo* investigations. To search for better EphA2 antagonists, we recently screened *in silico* a small collection of carboxylic acid derivatives available from Sigma-Aldrich (Saint Louis, MO, USA). A bunch of top-ranked compounds was purchased and tested in a wet binding assay. Among them, the 3β-hydroxy-Δ^5^-cholenic acid and the 4-(4-cyclopentylnaphthalen-1-yl)-4-oxobutanoic acid (Figure 1) were identified as inhibitors of the EphA2/ephrin-A1 interaction [18], with potency in the medium/high micromolar range.

The ability of *in silico* screening approaches to identify novel EphA2 receptor antagonists, prompted us to evaluate the performance of a variety of virtual screening (VS) approaches, starting from known chemical libraries of ready-to-ship compounds, typically used in VS campaigns. In the present work, we carried out a computational analysis where we compared the ability of standard ligand- and structure-based approaches to retrieve known EphA2 antagonists from different libraries of decoys. We applied shape-similarity and pharmacophore match techniques available in the Phase software package [19], and flexible ligand docking available in the Glide program [20]. The EphA2 antagonist UniPR129 and the ephrin-A1 peptide ligand were used as template structures to drive the search of actives by similarity and pharmacophore search. Docking runs were performed using the X-ray structure of EphA2/ephrin-A1 complex, recently reported in the literature [21]. The performance of each computational procedure was assessed by calculating the enrichment factor (EF), which is a measure of how many experimentally active compounds are found within a defined fraction of the ordered database relative to a random distribution [22].

## 2. Results and Discussion

**Figure 2:**
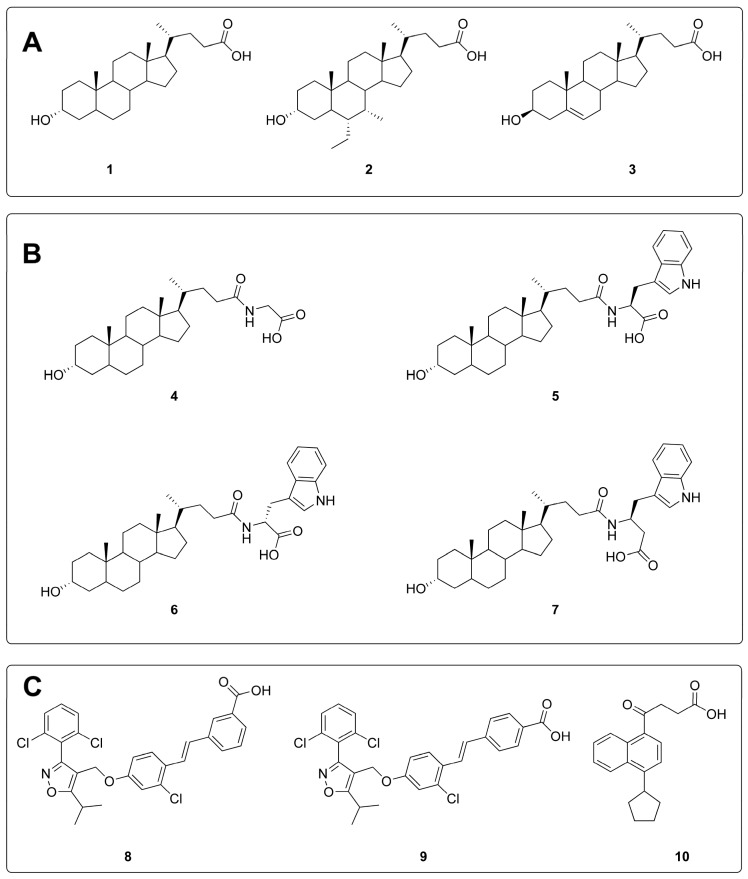
Known antagonists of the EphA2 receptor included in the VS analysis, divided according to their chemotype.

A retrospective evaluation of VS methods requires a set of active compounds and one or more chemical libraries of *bona fide* inactive compounds (decoys) [23]. In this study, the set of actives was composed by 10 inhibitors of the EphA2/ephrin-A1 interaction (Figure 2), representative of three main classes of available small-molecule antagonists of the EphA2 receptor. These were (**A**) bile acid analogues, including LCA (**1**) [12], INT-747 (**2**) [24] and 3β-hydroxy-Δ^5^-cholenic acid (**3**) [18]; (**B**) amino acid conjugates of LCA, with glycine (**4**), l-tryptophan (UniPR126, (**5**) d-tryptophan (**6**) [15], l-β-homo-tryptophan (UniPR129, **7**) [16]; and (**C**) three alkyl aryl carboxylic acids consisting of two stilbene derivatives, GW4064 (**8**) and PCM303 (**9**) [24] and the 4-(4-cyclopentylnaphthalen-1-yl)-4-oxobutanoic acid (**10**) [18]. As datasets of decoys, we selected two different chemical libraries of commercially available compounds, (i) the ChemDiv library [25] focused on protein–protein interaction (PPI) inhibitors and (ii) the complete ChemBridge library available at the ZINC website [26]. As the presence of a carboxylic acid group appeared to be a crucial feature to experimentally bind the EphA2 receptor [13], only compounds bearing at least one carboxylic acid group were selected from the ChemDiv PPI-focused database and from the ChemBridge library. The resulting libraries of carboxylic acids were further filtered to retain decoys with molecular properties (*i.e.*, MW, A log P, number of rotatable bonds) similar to those displayed by the known EphA2 antagonists here considered. The application of these filters was needed to avoid artificially high EF values caused by an excessively large structural diversity between active compounds and library decoys [27,28].

**Figure 3:**
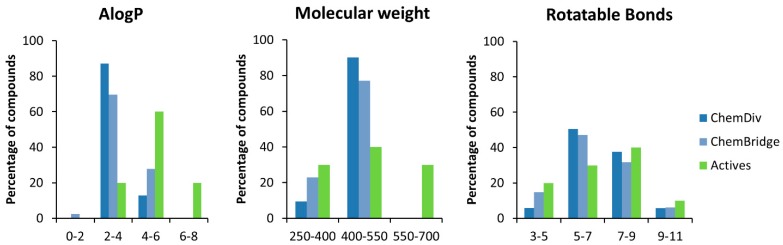
Histograms of selected molecular descriptors for the set of active compounds and the decoy datasets.

The EphA2 antagonists **1**–**10** possess physicochemical properties comparable to those shown by known PPI inhibitors reported in two independent studies reported by Sperandio [29] and Roche [30] research groups. Figure 3 shows the distribution of molecular properties for the decoys after the filtering procedure. Both ChemDiv and ChemBridge libraries do not fully covers the property range of the actives, at least in the case of lipophilicity (AlogP). Indeed, in the case of compounds **1**–**10**, AlogP distribution has a median of 4.9 units, whereas ChemDiv and ChemBridge libraries have AlogP distribution with medians of 3.2 and 3.5, respectively. Some small but significant differences can be detected among the distributions of the molecular weights, as the medians of MWs for the actives, ChemDiv and ChemBridge libraries are 487, 442 and 428, respectively. Finally, no significant disparities exist in terms of number of rotatable bonds, being the medians for the actives, ChemDiv and ChemBridge libraries of 7, 6 and 6, respectively. The differences in the molecular property profiles likely originates from the presence of several classes of compounds designed to bind “classical” targets (G-protein couple receptors, kinases and ion channels) in the commercial libraries [31]. Even after the applications of filters, which should realign the chemical space window of compound collections with the chemical requirements of PPI inhibitors [32], the composition of the ChemDiv and ChemBridge libraries remains biased toward compounds more hydrophilic and smaller than compounds present in the dataset of actives.

**Figure 4:**
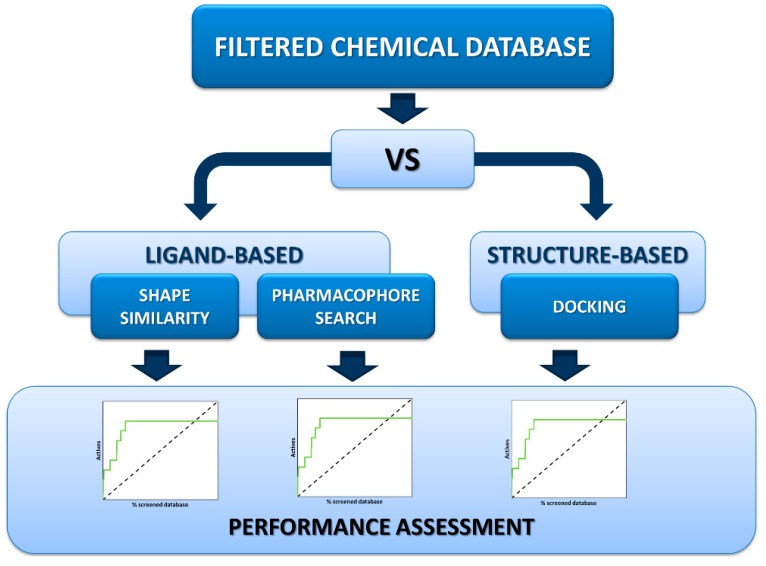
Virtual screening workflow applied in the present computational analysis.

We next performed VS runs on the filtered ChemDiv and ChemBridge libraries, applying both ligand-based and structure-based techniques according to the workflow reported in Figure 4. Given the “active” 3D conformation of one or more ligands, either obtained from X-ray, NMR or molecular modeling studies [33], 3D-similarity and pharmacophore searches can be used to virtually screen large chemical libraries at a reasonable computational cost [34]. Herein, we used the shape screening [35] and the pharmacophore search [36] modules available in Phase, which have been shown to give remarkable screening performances both in retrospective analysis [37] and hit-finding campaigns [38].

When the 3D-structure of a given target is known, ligands can be submitted to molecular docking (and scoring) to provide potential candidates for synthesis and experimental testing [39]. Due to the complexity of the energetics governing the protein–ligand association, the docking approach is generally the computationally most demanding VS procedure, although not necessarily the most effective in terms of performance [34]. Among the plethora of available docking software, we selected Glide program, considering the acceptable accuracy of its scoring functions [40,41], as well as the reasonable computational cost required by it for screening thousands of compounds even coupling docking runs to conformational search for each 3D entry of the chemical libraries (see Methods). For each chemical library, compounds (actives and decoys) were independently ranked according to their similarity score (shape screening), fitness score (pharmacophore search) or binding energy (docking simulations). Then, the performance of each VS run was assessed by calculating the enrichment factor (EF), which is a measure of how many actives are found within a defined fraction of the ordered database relative to a random distribution [22]. As only a small fraction of a given database is usually tested experimentally after a VS-based selection, EF factors were calculated considering the top 2% and 5% of the screened database (see Equation (1) in the Methods Section). In this way, it is possible to compare different VS methods on the basis of their ability to recognize actives as “early” as possible. By definition, EF can go from 0 to a maximum that depends (i) on the ratio between the number of actives and the total number of screened compounds and (ii) on the fraction of the database in which one may expect to find the actives. In our case, when the top 2% and 5% of the ranked database are selected, the maximum values of EF are 50 and 20, respectively.

### 2.1. Shape-Screening

Shape-based screening relies on the underlying assumption that the shape of a query molecule that is known to be active against a target of interest contains useful information that can help to retrieve other active molecules from a database of compounds. The shape screening protocol implemented in Phase performs an initial 3D alignment of each database compound to the query structure, which is followed by a scoring step, allows ranking all the entries, active compounds and decoys [35].

**Figure 5:**
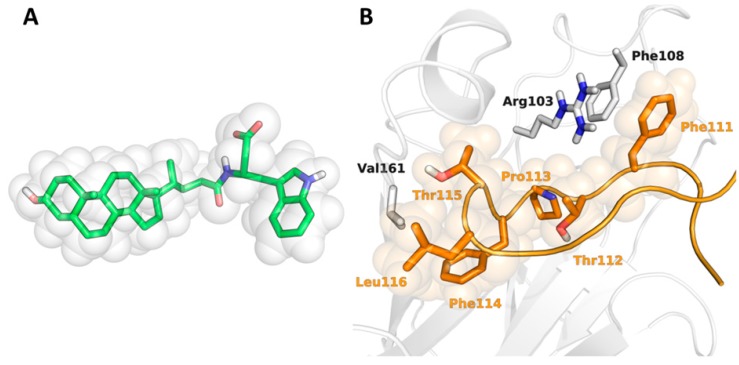
(**A**) 3D representation of UniPR129 in its minimum-energy conformation with its shape represented as a set of van der Waals spheres; and (**B**) X-ray structure (PDB: 3HEI) of EphA2 (white carbons) in complex with ephrin-A1 (orange carbons). The shape of the G-H loop of ephrin-A1 involved in the interaction with EphA2 is depicted with van der Waals spheres.

Here, we conducted shape screenings using the reference EphA2 antagonist UniPR129 in its global energy minimum conformation (Figure 5A) or the X-ray coordinates of G-H loop of ephrinA1 (Figure 5B) as query structures. The shape screenings were performed in three different modes [35]: (i) treating all atoms as equivalent during the alignment of a 3D entry to the template (*shape-only*); (ii) favoring alignments superimposing atoms of the same macromodel atom type (*mmod*); or (iii) favoring alignments superimposing the same pharmacophore features (*pharm*), according to a Phase standard definition.

Table 1 reports the EF values at 2% and 5% of the screened database for the shape-screening runs. When UniPR129 was used as template structure, shape-screening runs always yielded high EF_2%_ and EF_5%_ values, regardless of the rules employed to align each 3D entry to the template structures or of the type of chemical library. Indeed shape-screening runs performed in *mmod* and *pharm* modes were able to retrieve up to seven active compounds in the top 2% of both libraries, giving an EF_2%_ of 35.

**Table 1:** EF values calculated at 2% and 5% for the shape-screening simulations.

Template	Method	ChemDiv		ChemBridge	
		EF_2%_	EF_5%_	EF_2%_	EF_5%_
UniPR129	*shape-only*	25 (5)	14 (7)	25 (5)	14 (7)
	*mmod*	35 (7)	14 (7)	35 (7)	14 (7)
	*Pharm*	35 (7)	14 (7)	35 (7)	14 (7)
G-H loop	*shape-only*	20 (4)	10 (5)	15 (3)	6 (3)
	*mmod*	15 (3)	6 (3)	15 (3)	6 (3)
	*Pharm*	0 (0)	0 (0)	0 (0)	0 (0)

The number of actives found at 2% or 5% of the screened databases is reported in brackets.

When the shape screening was performed in *shape-only* mode the performance was slightly lower, yielding an EF_2%_ value of 25 for both libraries. Interestingly, visual inspection of the resulting hits at 5% of both screened databases, showed that *mmod* and *pharm* approaches were able to correctly identify only the steroidal derivatives (compounds **1**–**7**) as actives, classifying the remaining compounds (**8**–**10**) as false negatives. Conversely, the *shape-only* mode correctly retrieved at least one compound for chemical class (A, B or C) as active, being able to score compounds **1**–**2**, **4**–**7** and **9** within the 5% of both ranked databases.

The remarkable performance of the shape-screening approach is likely due to the low variability of the chemical structure of active compounds compared to the reference one. Indeed, as the shape-screening algorithm aligns molecules by selecting atom pairs with a similar local 3D environment, steroidal compounds **1**–**6** always obtain high shape similarity score when UniPR129 is used as query.

To challenge the shape-screening approach in a less biased scenario, we performed another shape-based VS using a reference query not derived from compounds **1**–**10**. More specifically, we selected the Phe111-Leu116 fragment of the G-H loop of ephrin-A1 as query structure. This small peptide fragment has been shown to be fundamental for the binding of ephrin-A1 ligand to EphA2 high affinity site [42] and thus its shape might be effective in retrieving active compounds. In this condition, the EFs were significantly lower than those obtained using UniPR129 as a query. In particular, the shape screenings performed on the G-H loop poorly performed in the case of the ChemBridge library, as only compounds **5**–**7** were found in early fractions of the screened database, yielding EF_2%_ and EF_5%_ values ranging from 0 to 15 and from 0 to 6, respectively (Table 1). That said, the shape screening was rather effective in identifying compounds **5**–**8** and **5**–**9** in the first 2% and 5%, respectively, of the screened ChemDiv library when the *shape-only* mode was applied. Accordingly, EF_2%_ and EF_5%_ values calculated for this run were 20 and 10, respectively.

**Figure 6:**
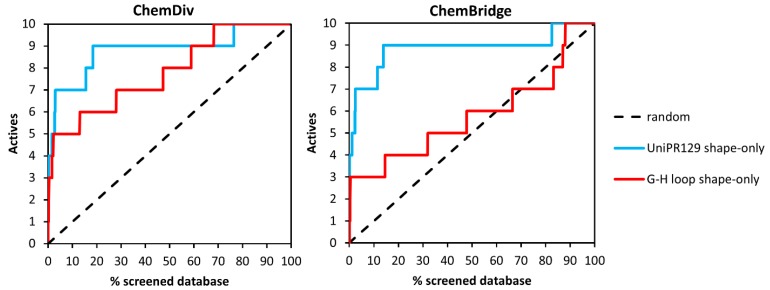
Enrichment curves obtained from shape screenings performed on ChemDiv **(left**) and Chembridge (**right**) libraries.

To analyze further the ability of the different shape-screening methods to correctly identify active compounds, we also calculated enrichment curves (Figure 6). These curves to evaluate how the fraction of actives recovered varies with the percent of the database screened, giving a measure of the overall performance of the VS run. Figure 5 shows enrichment curves obtained for ChemDiv and ChemBridge libraries applying the *shape-only* approach on both query structures, *i.e.*, UniPR129 and the G-H loop. The curves obtained for the ChemDiv remarkably deviates from the random selection distribution, confirming a fair ability of both VS runs to detect active compounds. Conversely, the screening based on ephrin-A1 G-H loop poorly performed for the ChemBridge library as indicated by the EF accumulation curve, which remains close to the random selection distribution. This modest performance could be to the unbalance between the molecular properties of the decoys, especially those of the ChemBridge library (median MW = 428, median Alog *p* = 3.5) and the molecular properties of the G-H loop of ephrin-A1 (MW =780; Alog *p* = 0.3).

### 2.2. Pharmacophore Search

**Figure 7:**
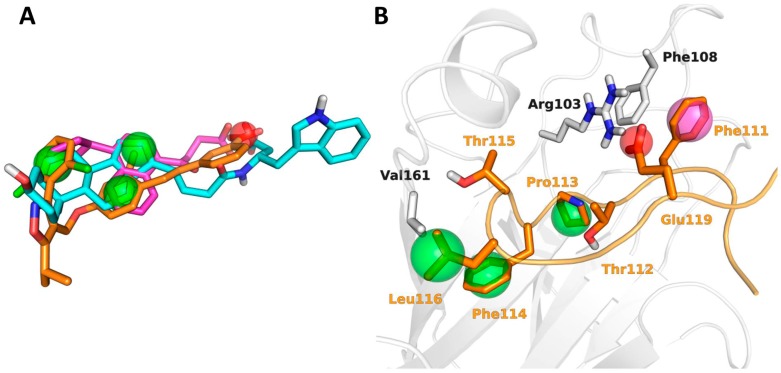
Pharmacophore models employed for VS. Pharmacophore sites are represented as spheres: hydrophobic (green), negatively charged (red), aromatic (magenta). (**A**) *Model I* superposed on the aligned structures of compounds **7** (cyan carbons), **8** (orange carbons), **10** (magenta carbons); (**B**) *Model II* represented on the crystallized conformation of the G-H loop of ephrin-A1 (orange carbons) within the structure of EphA2 (white cartoons and carbons).

Pharmacophore models represent the spatial arrangement of chemical features of one or more ligands that are needed for binding to a given receptor [43]. Ligand-based pharmacophore models can be applied when ligand’s active conformation is experimentally available or can be deduced from a set of structurally diverse ligands with known biological activity [44]. With this in mind, we used two independent sources of information to build pharmacophore models for VS applications. A first model was built starting from the common pharmacophore features shared by compounds **1**–**10** (*Model I*, Figure 7A), following the procedure described in the method section. A second pharmacophore model was built starting from the G-H loop of ephrinA1 (*Model II*, Figure 7B) through the application of a computational alanine-scanning procedure aimed at identifying a small group of ephrin-A1residues, critical for EphA2 binding, to be used as pharmacophore sites (see methods).

Figure 7 reported a graphical representation of the resulting pharmacophore models. *Model I* is constituted by four pharmacophore sites, three hydrophobic (H) and one negatively charged (N). These can be easily identified on the structure of some representative EphA2 antagonists, *i.e.*, UniPR129 (compound **7**), GW4064 (compound **8**) and 4-(4-cyclopentylnaphthalen-1-yl)-4-oxobutanoic acid (compound **10**), as reported in Figure 7A. *Model II* is a five point pharmacophore composed by three hydrophobic sites (H), corresponding to the side chains of Pro113, Phe114, and Leu116, one aromatic site (R), corresponding to the side chain of Phe111, and one negatively charged area (N) corresponding to Glu119 (Figure 7B).

Table 2 reports the EF values at 2% and 5% of the screened database for the pharmacophore VS runs. *Model I* always outperformed *Model II*, giving high EF_2%_ and EF_5%_ values regardless of the chemical library employed in the screening. The application of *Model I* gave EF values comparable to those obtained with the shape screening on UniPR129 as query structure with both chemical libraries. Indeed, pharmacophore *Model I* was able to retrieve up seven actives (compounds **1**, **3**, **4**–**7**, **8**) in the top 5% of both ChemDiv and Chembridge datasets of decoys, correctly identifying as active at least one compound for each chemical class.

**Table 2:** EF values at 2% and 5% obtained from pharmacophore searches.

Method	ChemDiv		ChemBridge	
	EF_2%_	EF_5%_	EF_2%_	EF_5%_
*Model I*	25(5)	14 (7)	30 (6)	14 (7)
*Model II*	15 (3)	6 (3)	15 (3)	6 (3)

The number of actives found at 2% or 5% of the screened databases is reported in brackets.

*Model II* gave lower performances compared to *Model I*, as the VS runs based on this pharmacophore were able to retrieve only three active compounds (**5**–**7**, all belonging to class **B** of actives) at 2% and 5% of both libraries. This modest result is term of EF is probably due to the rather high number and type of pharmacophore sites required to match *Model II*.

**Figure 8:**
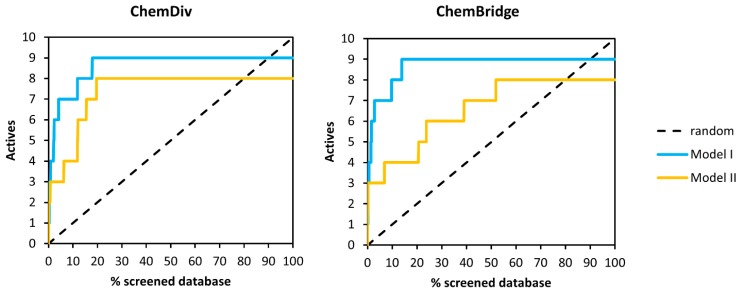
Enrichment curves obtained from pharmacophore searches performed on ChemDiv (**left**) and Chembridge (**right**) databases.

In addition, in the case of pharmacophore VSs enrichment, curves were built to have a measure of the overall performance of the two different models employed as a query. The enrichment curves reported in Figure 8 confirmed the trend observed with EF_2%_ and EF_5%_ with some of active compounds (*i.e.*, **2**, **10** for *Model I*,) and (*i.e.*, **2**, **4**, **8**–**10** for *Model II*) being found only at a high fraction of the screened databases. Some of the actives (**9** for *Model I* and **1** and **3** *for Model II)* could not be found at all by these VS procedures as they were discarded by the Phase scoring function.

### 2.3. Docking

**Figure 9:**
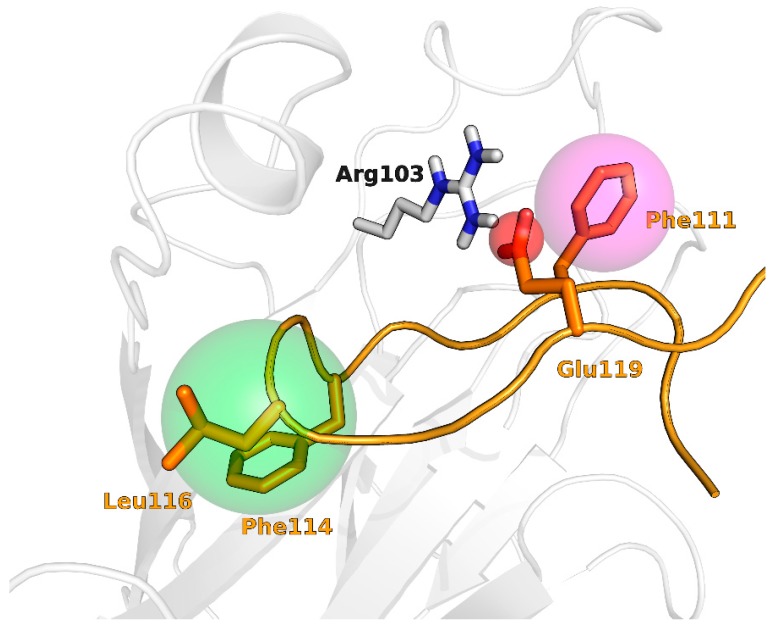
Constraints employed during knowledge-based docking runs**.** Constraints sites (hydrophobic, green; negatively charged, red; aromatic, magenta) are represented as spheres on the G-H loop of ephrin-A1 in complex with EphA2.

Docking is an established structure-based method to investigate the binding mode of small molecules into a well-defined protein pocket. The main advantage of docking is that it uses the structural information of protein binding site to drive the VS without being biased towards existing chemical classes of active compounds. In the present work, docking simulations were performed using the X-ray coordinate of the ligand binding domain of the EphA2 receptor, taken from its complex with ephrin-A1 ligand [21]. Docking runs were performed with Glide [20] using two different computational protocols. The first VS was based on a blind approach, while the second VS was a knowledge-based docking in which positional constraints were applied to select only those docking poses in which a given 3D-entry was occupying the binding region of the G-H loop of ephrin-A1. These positional constraints were a (i) hydrophobic region corresponding to the side chain of residues 114–116; (ii) an aromatic site placed on the side chain of Phe111; and (iii) a hydrogen bonding acceptor group to mimic the hydrogen bonding properties of Glu119 (Figure 9). To avoid a potential bias associated with the starting geometry of the ligand [45] a quick conformational search was performed on each 3D entry to retrieve ten different conformations to be used in docking calculations.

Table 3 reports the EF values at 2% and 5% of the screened database for the docking simulations. The blind docking protocol gave very modest EF_2%_ and EF_5%_ values close to 0 for both chemical libraries. On the contrary, the knowledge-based gave a much better performance with the ChemDiv library being able to identify three (**5**–**7**) and six (**1**, **4**–**7** and **10**) active compounds in the first 2% and 5% of the screened database, respectively, yielding EF_2_% and EF_5_% values of 15 and 12. The knowledge-based approach showed lower performances in the case of the Chembridge library, as only one (**6**) and three (**5**–**7**) active compounds were found at 2% and 5%, of the screened database, respectively.

**Table 3:** EF values at 2% and 5% obtained from docking simulations.

Method	ChemDiv		ChemBridge	
	EF_2%_	EF_5%_	EF_2%_	EF_5%_
*blind*	0 (0)	2 (1)	0 (0)	0 (0)
*knowledge-based*	15 (3)	12 (6)	5 (1)	6 (3)

The number of actives found at 2% or 5% of the screened databases is reported in brackets.

**Figure 10:**
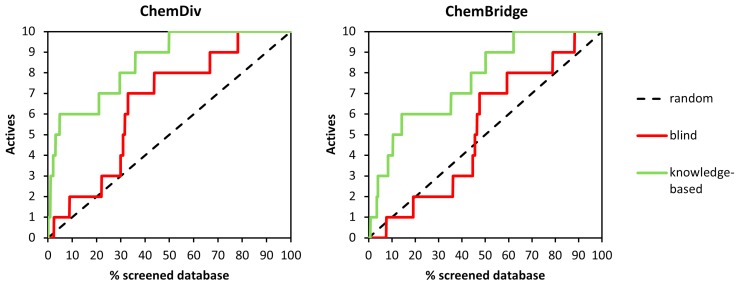
Enrichment curves obtained from docking simulations performed on ChemDiv (**left**) and Chembridge (**right**) libraries.

Figure 10 shows enrichment curves obtained with the docking protocols described previously. The knowledge-based procedure produced accumulation curves for both ChemDiv and ChemBridge libraries different from that corresponding to a random selection. Conversely, the blind docking runs gave lower VS performances, resulting in enrichment trends similar to that attainable with a random selection.

The unsatisfactory screening performances obtained with docking could be due to some issues related to the standard precision (SP) scoring function implemented in Glide. Glide scoring function estimates the free energy of binding with an empirical equation, which neglects an explicit treatment of entropic and desolvation contributions, two critical drivers of the interaction between large protein interfaces and compounds featured by rather high lipophilicity and molecular weight [46].

## 3. Materials and Methods

### 3.1. Database Preparation

Two libraries of 2D decoys, constituted by (i) a subset of the ChemDiv database [25] focused on PPI inhibitors and (ii) the whole ChemBridge library downloaded from Zinc database [26], were used in VS campaigns. Decoy molecules were selected to avoid biasing screening results. In particular, both libraries were initially filtered taking into account known structure-activity relationships (SARs) for known EphA2 antagonists. As the carboxylic group was found as a crucial feature EphA2 antagonist activity [13], two initial subsets of 3201 and 58000 compounds bearing at least one carboxylic acid group were selected from the ChemDiv PPI-focused database and from the ChemBridge library, respectively. A second filtering procedure aimed at retaining molecules with computed molecular properties close to those of compounds **1**–**10** (*i.e.*, MW 295–575, AlogP 2–8, rotatable bonds 4–9) was applied, The filtering procedure was conducted with the Ligfilter utility implemented in Maestro 9.6 [47]. This filtering procedure yielded 1646 and 2955 decoys for the ChemDiv PPI-focused and for the ChemBridge database, respectively. The 2D structures of the active compounds and of the selected decoys were converted in 3D structures with LigPrep 2.5 [48]. Only the most abundant ionization state at pH 7.4 was modeled according to calculations performed with Epik 2.2 [49]. The final databases contained a single conformation for each 3D entry as a throughout conformational analysis was performed prior to all VS calculations.

### 3.2. Shape-Screening

Shape screenings were conducted with Phase 3.7 [19]. The global minimum energy conformation of the EphA2 antagonist UniPR129 or the crystallographic coordinates of the G-H loop of ephrin-A1 extracted from the X-ray structure of the EphA2-ephrinA1 complex (PDB: 3HEI, [21]) were used as template structures. The global minimum-energy conformation of UniPR129 was found applying a conformational search performed using the mixed torsional/low-mode sampling approach (MCMM/LMOD) implemented in Macromodel [50], with default settings.

During the shape screenings, an “on-the-fly” conformational search was performed with ConfGen [51] on each molecule of the libraries, generating up to 100 conformers that were treated as a set of hard spheres and flexibly superposed atom-to-atom on the template molecules. A shape-similarity score was thus calculated from the overlap between hard-spheres volumes of superposed atoms. The shape-screening algorithm can compute a similarity metric regardless of the atom types of the superposing atoms (*shape-only*), or it can compute a similarity score only for those superimpose atoms sharing the same macromodel atom type (*mmod*) or the same pharmacophoric features (*pharm*) [35]. The screened databases were ranked according to the shape-similarity score, and only the best scored conformer for each compound was retained.

### 3.3. Pharmacophore Models Building

Phase 3.7 was used to generate pharmacophore models starting from the 3D structures of compounds **1**–**10** (*Model I*) or from the X-ray structure of the ephrin-A1 ligand (*Model II*). For the building of *Model I*, compounds **1**–**10** were submitted to a conformational analysis using Macromodel. The conformational searches were carried out with the mixed torsional/low-mode sampling approach (MCMM/LMOD) using default settings retaining up to 30 conformers for each ligand. The pharmacophore sites were assigned to each conformer using the default set of features implemented in Phase. Several pharmacophore hypotheses were developed starting from the set of conformers identified for compounds **1**–**10** by the MCMM/LMOD procedure and subsequently ranked according to the Phase scoring function. Only hypotheses based on three hydrophobic (H) and one negatively charged (N) features were considered as they gave pharmacophore models shared by at least 8 active compounds. Among the different HHHN pharmacophore models, the one having the highest Phase score was retained and used for VS calculations.

**Figure 11:**
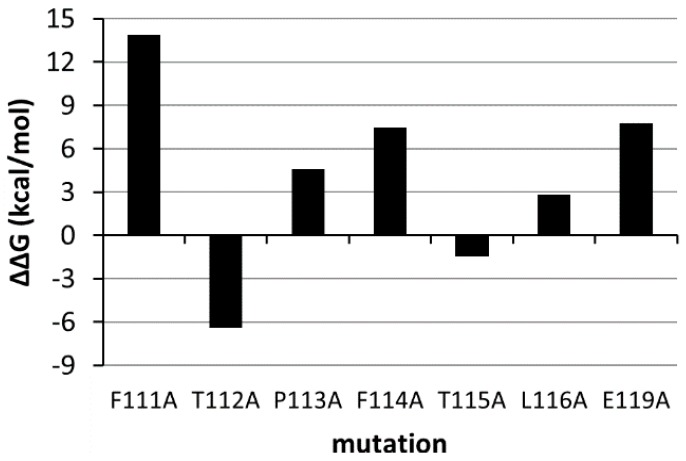
Estimated ΔΔG after mutation in alanine for residues 111–116 and 119 of the G-H loop of ephrin-A1.

The construction of *Model II* involved the selection of the most critical residues of the G-H loop for EphA2 binding (residues 111–119). To this aim, we performed a computational alanine-scanning [52] procedure using the MM-GBSA method implemented in Prime 3.4 [53] in combination with energy minimization. During this procedure, only the G-H loop of ephrin-A1 as well as all EphA2 residues located 8 Å far from it were treated flexibly. Pharmacophore features were then mapped on the residues that produced an increase in estimated free energy of at least 2 kcal/mol once mutated in alanine (Figure 11). The type of pharmacophore feature mapped on the each selected residue was selected on the basis of the properties of its side chain. The final pharmacophore model was constituted by five sites, three of which were hydrophobic (Pro113, Phe114, Leu116), one negatively charged (Glu119) and one aromatic (Phe111).

### 3.4. Pharmacophore Search

For each active and decoy compound, an “on-the-fly” conformational search was performed during the pharmacophore screening with ConfGen, generating up to 1000 conformers that were aligned on models *I* and *II* and subsequently ranked according to the Phase Fitness score [36]. For *Model I*, the criteria of hit retrieval was the match of all pharmacophore sites with a distance tolerance of 2 Å. For *Model II*, the criteria of hit retrieval was the match of at least two hydrophobic (Pro113, Leu116) and the negatively charged sites (Glu119) with a distance tolerance of 3 Å. The weights applied to the components of the Phase scoring function were left unchanged.

### 3.5. Docking

The EphA2/ephrin-A1 complex crystal structure (PDB: 3HEI, [21]) was first processed with the protein preparation tool of Maestro [47] which added missing hydrogen atoms and optimized the overall hydrogen bonding network by adjusting the tautomerization and protonation states of histidine, aspartate and glutamate residues and by sampling the orientation of water hydrogens and of the side chains of polar amino acids. The resulting complex structure was submitted to a restrained minimization applying the OPLS2005 force field [54] to a RMSD value of 0.3 Å calculated on protein heavy atoms. The ephrin-A1 ligand and all water molecules, which did not form key hydrogen bond interactions within the EphA2 binding site, were deleted prior to docking calculations. The docking grid was centered in a region delimited by Arg103, Phe156 and Arg159, which encompasses the main hydrophobic channel of the EphA2 receptor. Dimensions of enclosing and bounding boxes were set to 30 Å and 10 Å for each side, respectively. Prior to docking calculations, each molecule of the database was subjected to a conformational search (with MCMM/LMOD, using default settings) to generate up to 10 ligand conformations, which were subsequently submitted to docking simulations. Docking runs were performed with Glide in Standard Precision (SP) mode, both in presence and in absence of constraints involving (i) an hydrophobic region corresponding to the side chain of residues 114–116; (ii) an aromatic site placed on the side chain of Phe111; and (iii) a hydrogen bonding group to mimic the hydrogen bonding properties of Glu119. Five docking poses were collected for each compound and only the top ranked solution in terms of Docking Score was retained.

### 3.6. Performance Assessment

Several metrics for assessing VS performance have been proposed [55]. As in a real-case scenario only a small fraction of a database could be tested experimentally, it is often important to recognize actives in the first fraction of the ranked database. In the present work, we used the EF as screening metric, which is a measure of how many actives are found within a defined fraction of the ordered database relative to a random distribution [22]. A percentage threshold x% is chosen to define the fraction of interest of the ranked database and the number of actives found within this percentage is compared to the number of actives one would expect to find from a random selection, using the following equation:

(1) $${\mathrm{EF}}_{\mathrm{x\%}}=\frac{\mathrm{act/n}}{\mathrm{ACT/N}}$$

where *act* is the number of actives retrieved in the first *n* positions of the database and ACT is the total number of actives included in the database of N compounds. In this study, EFs were calculated at 2% and 5% of the total database screened and enrichment curves were built by plotting the number of actives found as a function of the percentage of the screened database.

## 4. Conclusions

We performed a retrospective analysis of different ligand- and structure-based screening methods to find a VS protocol that can be effectively applied in prospective drug design campaigns aimed at identifying novel EphA2 antagonists.

A comparison of EF values obtained from the different VS methodologies showed that ligand-based approaches outperformed the structure-based ones, with the shape similarity showing the best performances. Nevertheless, few pitfalls can be identified for each ligand-based method. Indeed, the shape similarity approach resulted strongly affected by the choice of the reference structure, giving artificially high enrichment factors when the query molecule was structurally related to the active compounds. Moreover, results showed that only the shape-similarity screening in *shape-only* mode and pharmacophore search with *Model I* were able to retrieve at least one active compound for each chemical class.

Despite its higher computational cost, docking-based VSs offered rather poor performances. These could be ascribed to (i) a lack of an exhaustive conformational search protocol and/or (ii) the scoring function, which may not be properly calibrated to model ligand accommodation into wide and solvent exposed surfaces such as those involved in PPI, leading to a remarkable increase in the number of false positives [56]. In this study, we tried to overcome the potential bias associated with the starting ligand geometry applying a conformational search on each molecule of the databases before docking calculations [57]. However, the docking process still gave poor performances, indicating that Glide SP scoring function was not able to distinguish actives from decoys, even after the application of positional constraints biasing the position of each docked ligand toward a pose resembling that one assumed by the G-H loop of ephrin-A1 in its X-ray structure with EphA2 [21]. Blind docking runs performed with AutoDock Vina [58] produce enrichment curves comparable to those obtained with Glide (data not shown), confirming the trend that shape screening and pharmacophore search outperform docking runs in the case of the EphA2 receptor.

That said, a “combined” approach taking into account both target- and ligand-based information could represent a valuable computational strategy to search for novel active compounds [59]. In our case, the crystallographic-derived pharmacophore model (*Model II*) emerges as a reasonable compromise between structure- and ligand-based methodologies in term of performance and computational cost. Indeed, despite not being able to identify all the active compounds in an early stage of the screening, pharmacophore *Model II* retrieved both steroidal and non-steroidal compounds, suggesting that it might be exploited for scaffold-hopping campaigns aimed at searching for novel structurally unrelated EphA2 antagonists.
